# Hypertension and Associated Risk Factors among Children with Intellectual Disability: A Cross-Sectional Study

**DOI:** 10.3390/nu14153127

**Published:** 2022-07-29

**Authors:** Yan Sun, Rashmi Supriya, Yang Gao, Dan Tao, Siyue Yu, Aiwei Wang, Hardaway Chun-Kwan Chan, Xiaoting Ou, Jingjing Wang, Julien S. Baker

**Affiliations:** 1 Department of Sport, Physical Education and Health, Hong Kong Baptist University, Hong Kong 999077, China; 19482302@life.hkbu.edu.hk (Y.S.); rashmisupriya@hkbu.edu.hk (R.S.); 17482062@life.hkbu.edu.hk (A.W.); hardawayc@life.hkbu.edu.hk (H.C.-K.C.); 21481059@life.hkbu.edu.hk (X.O.); jsbaker@hkbu.edu.hk (J.S.B.); 2 Centre for Health and Exercise Science Research, Hong Kong Baptist University, Hong Kong 999077, China; 3 Department of Government and International Studies, Hong Kong Baptist University, Hong Kong 999077, China; 21483132@life.hkbu.edu.hk; 4 JC School of Public Health and Primary Care, The Chinese University of Hong Kong, Hong Kong 999077, China; yusiyuey@gmail.com; 5 College of Physical Education, Yangzhou University, Yangzhou 225012, China; 6 Scientific Conditioning Centre, Hong Kong Sports Institute, Hong Kong 999077, China; 7 Mass Sports Research Center, China Institute of Sport Science, Beijing 100062, China; wangjingjing@ciss.cn

**Keywords:** hypertension, children, intellectual disability, risk factor, cross-sectional study

## Abstract

To investigate the prevalence of hypertension and associated risk factors in Chinese children with intellectual disability, a cross-sectional study was conducted in a sample of 558 children with intellectual disability aged 6–18 years in Hong Kong, and 452 (81.0%) with valid data were included in the data analysis. Blood pressure was measured according to a standard protocol. Hypertension was defined using the age-, gender-, and height-specific classification criteria recommended by the 2018 Chinese Guidelines for Children. Multivariate and hierarchical logistic regression was fitted to examine the associations of hypertension with potential risk factors. Overall, 31.4% of the participants were classified as having hypertension. Obese children were more likely to develop hypertension than non-obese children (adjusted OR = 2.77, 95% CI: 1.28, 5.99, *p* = 0.010). A paternal education of college or above and a paternal occupation of clerks, sales representatives, and workers were also associated with an increased risk of hypertension. The prevalence of hypertension is high among Chinese children with intellectual disability. Obesity was the strongest risk factor. Further longitudinal studies are warranted to confirm our findings. Nevertheless, preventions against obesity are promising to receive doubled benefits in reducing both obesity and hypertension, given its strong relationship with hypertension in this special population.

## 1. Introduction

Pediatric hypertension has been an alarming public health issue [1,2]. Globally, several meta-analyses have reported a range of pooled prevalence rates of pediatric hypertension from 4.0% to 9.8%, with marked differences being observed in the reported prevalence across individual studies [1,3,4,5]. In China, a nationwide survey in 2010 demonstrated that 18.4% of children were hypertensive [6]. A similar prevalence rate was also observed in Hong Kong (boys: 15.1% vs. girls: 13.8%) [7].

Well-documented evidence supports that hypertension in childhood would continue into adulthood and translate into an increased burden of non-communicable diseases and a high risk of mortality in later life [8,9,10,11]. In addition, around 30–40% of children with hypertension have early signs of end-organ damage [12,13]. Therefore, the detection, evaluation, and intervention of elevated blood pressure in early life are essential measures to prevent, control, and manage hypertension and lower the risks for other health conditions in adulthood.

Children with intellectual disability (ID) may be more vulnerable to hypertension than their typically developing peers. Previous studies—though not many—observed prevalence rates of hypertension ranging from 11.7% to 21.1% [14,15,16]. Wyszyńska et al., in their study, further revealed a dramatically higher prevalence rate in children with ID compared to their typically developing peers (18.5% vs. 5.8%) [16]. A large body of research on children without ID indicated that body weight status, diet and nutrition, sleep, physical activity (PA), and some family factors were associated with hypertension [17,18]. However, the problem size and risk factors of hypertension in children with ID are still unclear. Our previous study revealed a higher prevalence of overweight and obesity in children with ID than their typically developing counterparts (31.3% vs. 19.9%) [19,20]. Children with ID were reported with less PA, more sedentary time, and a less healthy diet [14,21], as they may face more difficulties in understanding and adopting healthy lifestyles [22,23]. This paper aimed to investigate the prevalence of hypertension and its risk factors in a sample of Chinese children with ID to add evidence to the knowledge gap, which is imperative for developing effective interventions against hypertension in this special pediatric population.

## 2. Methods

### 2.1. Study Design and Participants

This was a cross-sectional study conducted from June to November 2015, targeting children with mild and moderate ID (intelligence quotient ranging from 50–69 for mild ID and 35–49 for moderate ID) [24] in special schools in Hong Kong. All 31 special schools of this type were invited to participate in the study, and 12 of them agreed to participate. Then, all eligible children in the 12 participating schools were invited to participate in the study. Written informed consent from their parents or guardians was obtained. The study was ethically approved by the Research Ethics Committee of Hong Kong Baptist University (Ref. No. FRG1/13-14/067).

### 2.2. Measures and Definitions

#### 2.2.1. Objective Measurements

Children’s right-arm blood pressure (systolic and diastolic blood pressure in mmHg) in a sitting position was measured after a resting time of at least 5–10 min in a quiet room at school in the morning time by trained investigators using a digital automatic blood pressure monitor (OMRON M6 Compact (HEM-7000-E)) with an adapted cuff, following a standard instruction manual [25]. Two measurements were performed at a 2 min interval. If the two blood pressure values differed by more than 5 mmHg, a third measurement was followed. The average of the last two measurements was used for data analysis. Hypertension was defined as systolic and/or diastolic blood pressure equal to or above the 95th percentile for age, gender, and height, as recommended in the 2018 Chinese Guidelines for Prevention and Treatment of Hypertension for Children [26].

Children’s height (in cm) and weight (in kg) were measured without shoes and heavy clothes, following a standard protocol, before the measurement of blood pressure. Obesity was classified according to the international age- and gender-specific body mass index (BMI) cut-offs recommended by Cole et al. [27].

#### 2.2.2. Subjective Measurements

A self-administered questionnaire was adopted to collect information on the background characteristics of children and parents, as well as children’s health-related behaviors. The details have been reported elsewhere [19].

The background characteristics of children included age, gender, birthplace, ID level, and comorbidities (e.g., autism, attention deficit hyperactivity disorder (ADHD), down syndrome, and epilepsy). Children’s health-related behaviors included PA, sedentary behavior, sleep duration, and dietary intake. The 24-hour movement guidelines for children involving sleep were adopted to classify the average sleep duration into two groups (sufficient vs. insufficient) [28]. Considering that children with ID have difficulties understanding and completing questionnaires independently, while their parents may have insufficient knowledge about children’s PA and diet at school, we combined parental proxy reporting with self-reporting to improve the accuracy of the information obtained [29].

Parental characteristics regarding their education, occupation, and marital status, as well as their height (in cm) and weight (in kg), were also self-reported with the questionnaire. Using the classification criteria for Chinese adults, parental BMIs equal to or above 28 kg/m^2^ were categorized as obese [30].

### 2.3. Questionnaire Administration and Quality Control

We launched a pilot survey to examine the feasibility of the procedures and the reliability of the questionnaire. The average Kappa was 0.71, indicating that the test-retest reliability of the questionnaire is acceptable [19].

### 2.4. Statistical Analysis

Participants (aged 6–18 years) with blood pressure measurements were included in the data analysis. Continuous and categorical variables were presented with the mean and standard deviation (SD) and the number and percentage, respectively. Chi-square tests were employed to examine the differences in the background characteristics and children’s health-related behaviors between the hypertension and non-hypertension groups. Univariate and multivariate logistic regressions were fitted to assess the relationship of hypertension with the background characteristics and children’s health-related behaviors. The unadjusted odds ratio (OR_unadj._) and adjusted odds ratio (OR_adj._) were presented, along with their 95% confidence intervals (95% CIs). In the multivariate analyses of the background characteristics, age and gender were adjusted for—except for gender, which was adjusted for age only. The background variables with *p* < 0.10 for OR_adj._ were additionally adjusted for in the multivariate logistic models of the children’s health-related behaviors, given that the background characteristics may play a role in the associations between hypertension and children’s health-related behaviors. Finally, hierarchical logistic regression was performed to examine the associations of hypertension with multiple studied variables in a full model [19], where gender and age were forcedly entered in Block 1; the background characteristics with *p* < 0.10 for OR_adj._ were forward selected in Block 2; the health-related behaviors with *p* < 0.10 for OR_adj._ were forward selected in Block 3. The model was set with *p* = 0.10 and *p* = 0.15 as the entry and removal criteria, respectively. Statistical analysis was performed using IBM SPSS Statistics 25. Statistical significance was deemed at two-tailed *p* < 0.05.

## 3. Results

A total of 558 participants returned the consent forms, indicating a response rate of 26.8%. Children aged over 18 years and those without valid blood pressure measurements were excluded, leaving 452 participants in the data analysis. The mean age of the participants was 11.78 years (SD: 3.50 years), 68.8% of them were male, and 73.1% were with mild ID (Table 1). Of the 452 participants included, 142 were defined as having hypertension, indicating a prevalence rate of 31.4%. The prevalence of hypertension was significantly higher in obese children and those with a paternal occupation of clerks, sales representatives, and workers.

Table 2 shows the distribution of hypertension by health-related behaviors. The results from the chi-square tests revealed no significant difference in any of the behavior variables studied. Children who consumed at least two portions per day of fruit and those who consumed at least three portions per day of vegetables were with a *p* < 0.10 and were therefore adjusted for in the further multivariate analyses.

Table 3 presents the associations between background characteristics and hypertension in children with ID before and after adjustment for age and gender. Obese children (OR_adj._: 3.21, 95% CI: 1.66–6.18, *p* < 0.001) and paternal occupations with clerks, sales representatives, and workers (OR_adj._: 1.71, 95% CI: 1.10–2.68, *p* = 0.018) were significantly associated with a higher risk of hypertension.

We examined the associations between health-related behaviors and hypertension in children with ID before and after adjustment for children’s gender, age, and background characteristics with *p* < 0.10 for OR_adj._, including obesity, marital status, and paternal occupation (Table 4). However, no significant associations were observed.

Ultimately, the results of the hierarchical logistic regression showed that obesity, paternal occupation, and paternal education were significantly associated with hypertension, with obesity being the strongest risk factor (OR_adj._: 2.77, 95% CI: 1.28–5.99, *p* = 0.010) (Table 5). No behavior variables met the entry criterion of *p* < 0.10 (Table 4) for selection in Block 3. Instead, we forward selected all of them in Block 3 regardless of their *p* values; but none were kept in the final model according to the variable selection criteria of *p* < 0.10 for entry and *p* > 0.15 for removal.

## 4. Discussion

This cross-sectional study highlights an alarmingly high prevalence of hypertension (31.4%) in Chinese children with ID. Children’s obesity, a paternal education of college or above, and a paternal occupation of clerks, sales representatives, and workers were identified as predisposing factors contributing to hypertension, whereas no significant associations were found between children’s health-related behaviors and hypertension in the studied population.

Our study found about one-third of the participants (31.4%) were hypertensive, which is higher than the rates found in previous studies on children with ID (range: from 11.7% to 21.1%) [14,15,16]. Differences in blood pressure measurements, cut-off points for the classification of hypertension, the characteristics of study populations (e.g., regions, ages, ID levels), and investigation years may contribute to the varied prevalence rates reported across studies [32,33]. For example, a study among 856 Chinese adolescents with ID (aged 15–18 years) revealed a relatively low prevalence of hypertension (11.7%), using the criteria of hypertension for adults (i.e., systolic blood pressure ≥ 140 mmHg or diastolic blood pressure ≥ 90 mmHg) [14]. The cut-off points for adults are higher than those for children, which may have led to the misclassification and underestimation of the prevalence of hypertension in that study. In addition, Wyszyńska et al. (2017) reported a lower prevalence (18.5%) in Polish children with ID, which may be attributable to regional differences, as a higher prevalence was found in typically developing Chinese children than in Caucasians [16,34].

Although there is a paucity of research investigating the prevalence of hypertension in children with ID, ample studies in typically developing children have reported prevalence rates with a large variation (ranged from 2.2% to 26.4%) [1,5]. It is suggested that the number of visits may make a major contribution to the wide range [35]. For example, a meta-analysis with no limit on the number of visits reported a pooled prevalence of 11.2% [34], which is nearly triple that (4.0%) found in another meta-analysis that included individual studies with at least three visits only [1]. In addition, unlike adults, there is no universal guideline for children. Diverse classification criteria were adopted across studies, which may also play a role in the varied hypertension prevalence. Zhou et al. (2022) compared three commonly used guidelines in 28,715 Chinese adolescents (aged 15–17 years) and found that the 2018 Chinese Guidelines for Prevention and Treatment of Hypertension for Children, which were used in our study, generated the highest prevalence rate of 24.4% [36]. The other two values were 18.6% for the 2017 American Association of Pediatrics Clinical Practice Guideline and 3.5% for the 2018 Chinese Guidelines for Prevention and Treatment of Hypertension for Adults.

Despite the above-mentioned heterogeneity in estimating the problem size of hypertension, we found a higher prevalence rate (31.4%) in children with ID in comparison with their typically developing peers (24.4%), as reported by Zhou et al. (2022) using the same classification criteria for hypertension. Research directly comparing the prevalence between children with and without ID is scarce. A Polish study involving the two populations reported a prevalence of 18.5% in children with ID (aged 7–18 years) and a prevalence of 5.8% in their counterparts without ID [16], which is in line with our findings.

As expected, this study demonstrated that obese children with ID were nearly three times (OR_adj._: 2.77, 95% CI: 1.28–5.99, *p* = 0.010) more likely to develop hypertension than their non-obese peers, indicating that obesity was the strongest risk factor. Consistently, previous studies have shown that children with obesity (vs. healthy body weight) are at an elevated risk of hypertension (range of ORs: 2.5–7.88) [21,37,38,39]. Possible mechanisms of obesity-associated hypertension included the activation of the sympathetic nervous system, insulin resistance and inflammation, an altered vascular profile, oxidative stress during obesity, etc. [40]. Hence, maintaining a healthy body weight is imperative for the prevention of hypertension in this population. Thus, interventions for obesity may induce doubled benefits for both obesity and hypertension.

This study found that children whose fathers were clerks, sales representatives, and workers (vs. administrators and professionals) and those with a paternal education of college or above (vs. junior secondary and below) were more likely to be hypertensive. No previous studies on this special population addressed these associations. The findings in general pediatric populations are also mixed [41,42,43,44]. Thus, there is a research gap in the role of these parental factors in childhood hypertension. Further studies are thus warranted, particularly for children with ID.

Against our hypothesis, our results showed no significant associations between PA, sedentary behavior, sleep, and hypertension. There is a scarcity of literature examining these associations in children with ID. Inconsistently, a previous study on children with ID reported that those with low PA level (vs. moderate PA level) had more than four times the risk of hypertension; those with a long sedentary time (vs. <2 h/day) were two times more likely to develop hypertension [21]. To our knowledge, no previous study reported the role of sleep in children with ID. Nevertheless, differences in the measurements and cut-off points for grouping the behaviors may at least partially result in the discrepancy of the findings. In addition, the small portion of active participants in our study (7%) may have led to a decreased statistical power in observing an actual significant association. Studies on typically developing children also reported conflicting results. Some studies reported significant associations with PA [37,45], sedentary behaviors [46], and sleep [47], while others did not [37,48,49]. Future studies are needed to establish the causal relationship between movement behaviors and hypertension in children, regardless of ID level.

We failed to observe significant associations between the studied dietary behaviors and hypertension. To our knowledge, this study is the first of its kind to examine their relationship in children with ID. For typically developing children, no consistent conclusions can be drawn on this issue [37,50,51]. Thus, longitudinal studies are promising to provide robust conclusions on the causal associations between dietary behaviors and hypertension in childhood. Though findings on the role of diet in childhood hypertension are mixed, given that dietary preferences are formed in childhood, interventions and strategies to promote a diet rich in fruits, vegetables, whole-grains, and low-fat dairy, along with a lower consumption of sodium, should be taken as early as possible to tackle hypertension and other related diseases [52].

Several limitations should be considered in this study. First, due to the cross-sectional study design, a causal inference cannot be made, and true associations may have been distorted or obscured. Second, although the questionnaires were completed by the parents with the children’s assistance, recall bias and reporting bias may still exist; thus, objective measurements, if possible and feasible, are recommended (e.g., using accelerometers to measure PA). Third, although some studies in general pediatric populations identified high sodium intake as a risk factor for hypertension [37,53,54], we failed to examine this relationship, as, by then, we did not have a validated Chinese questionnaire involving this item. Last but not least, although parental hypertension may contribute to pediatric hypertension [55], we did not ask about this information, as, in Hong Kong, near half of hypertension cases (47.5%) are undiagnosed [56]. Thus, self-reported hypertension may result in serious misclassification, which may further lead to a biased conclusion on their relationship.

## 5. Conclusions

The prevalence of hypertension is high among Chinese children with ID. Obesity, paternal education, and paternal occupation were identified as risk factors, with obesity being the strongest one. Longitudinal studies are warranted to confirm our findings. Nevertheless, there is an urgent need for interventions to reduce hypertension in this special population, while school-based programs, policies, and strategies regarding health promotion and involving both children and parents are promising. Blood pressure monitoring can be introduced as part of routine physical examinations for school children, which would identify and control hypertension at an early stage and lead to a reduced disease burden of hypertension.

## Figures and Tables

**Table 1:** Distribution of hypertension by background characteristics in children with intellectual disability (*N* = 452).

	*n* (%)	Non-Hypertension *n* (%)	Hypertension ^a^ *n* (%)	*p*-Value
Children’s Characteristics				
Gender				0.304
Male	311 (68.8)	218 (70.1)	93 (29.9)	
Female	141 (31.2)	92 (65.2)	49 (34.8)	
Birthplace				0.975
Hong Kong	388 (85.8)	266 (68.6)	122 (31.4)	
Others	64 (14.2)	44 (68.8)	20 (31.3)	
ID level				0.147
Mild (IQ: 55–69)	320 (73.1)	224 (70.0)	96 (30.0)	
Moderate (IQ: 35–54)	118 (26.9)	74 (62.7)	44 (37.3)	
Autism				0.401
No	193 (42.9)	128 (66.3)	65 (33.7)	
Yes	257 (57.1)	180 (70.0)	77 (30.0)	
ADHD				0.467
No	296 (65.8)	206 (69.6)	90 (30.4)	
Yes	154 (34.2)	102 (66.2)	52 (33.8)	
Down Syndrome				0.824
No	410 (91.1)	280 (68.3)	130 (31.7)	
Yes	40 (8.9)	28 (70.0)	12 (30.0)	
Epilepsy				0.780
No	416 (92.4)	284 (68.3)	132 (31.7)	
Yes	34 (7.6)	24 (70.6)	10 (29.4)	
Obesity ^b^				0.000 *
No	408 (90.9)	289 (70.8)	119 (29.2)	
Yes	41 (9.1)	18 (43.9)	23 (56.1)	
**Parental characteristics**				
Paternal education				0.285
Junior secondary and below	136 (32.1)	100 (73.5)	36 (26.5)	
Senior secondary	157 (37.0)	102 (65.0)	55 (35.0)	
College or above	131 (30.9)	91 (69.5)	40 (30.5)	
Maternal education				0.779
Junior secondary and below	145 (33.4)	97 (66.9)	48 (33.1)	
Senior secondary	182 (41.9)	124 (68.1)	58 (31.9)	
College or above	107 (24.7)	76 (71.0)	31 (29.0)	
Paternal occupation				0.047 *
Administrators and professionals	161 (35.6)	122 (75.8)	39 (24.2)	
Others (clerks, sales representatives, and workers)	254 (56.2)	165 (65.0)	89 (35.0)	
Missing	37 (8.2)	23 (62.2)	14 (37.8)	
Maternal occupation				0.424
Housewives	217 (50.6)	143 (65.9)	74 (34.1)	
Administrators and professionals	80 (18.6)	58 (72.5)	22 (27.5)	
Others (clerks, sales representatives, and workers)	132 (30.8)	94 (71.2)	38 (28.8)	
Parental marital status				0.080 ^†^
Married/cohabiting	359 (82.5)	242 (67.4)	117 (32.6)	
Divorced/separated/widowed	76 (17.5)	59 (77.6)	17 (22.4)	
Paternal obesity ^c^				0.905
No (BMI < 28 kg/m^2^)	320 (70.8)	221 (69.1)	99 (30.9)	
Yes (BMI ≥ 28 kg/m^2^)	56 (12.4)	37 (66.1)	19 (33.9)	
Missing	76 (16.8)	52 (68.4)	24 (31.6)	
Maternal obesity ^c^				0.872
No (BMI < 28 kg/m^2^)	381 (84.3)	262 (68.8)	119 (31.2)	
Yes (BMI ≥ 28 kg/m^2^)	28 (6.2)	18 (64.3)	10 (35.7)	
Missing	43 (9.5)	30 (69.8)	13 (30.2)	

Missing data < 6% were not presented in this table, which were also not counted when calculating percentages [31]. ^a^ Hypertension was defined as systolic and/or diastolic blood pressure equal to or above the 95th percentile for age, gender, and height recommended in the 2018 Chinese Guidelines for Prevention and Treatment of Hypertension for Children [26]. ^b^ Child obesity was defined by age- and gender-specific cut-off points of BMI recommended by Cole [27]. ^c^ Parental obesity was defined as BMI ≥ 28 kg/m^2^ for Chinese adults [30]. † *p* < 0.10; * *p* <0.05. Abbreviations: ID, intellectual disability; IQ, intelligence quotient; ADHD, attention deficit hyperactivity disorder; BMI, body weight index.

**Table 2:** Distribution of hypertension by health-related behaviors in children with intellectual disability.

	*n* (%)	Non-Hypertension *n* (%)	Hypertension ^a^ *n* (%)	*p*-Value
Physical activity				0.934
MVPA ≥ 60 min/day	31 (7.0)	21 (67.7)	10 (32.3)	
MVPA < 60 min/day	409 (93.0)	280 (68.5)	129 (31.5)	
Sedentary behavior				0.710
<4 h/day	221 (53.4)	152 (68.8)	69 (31.2)	
≥4 h/day	193 (46.6)	136 (70.5)	57 (29.5)	
Sleep duration				0.210
Sufficient ^b^	264 (62.3)	187 (70.8)	77 (29.2)	
Insufficient ^b^	160 (37.7)	104 (65.0)	56 (35.0)	
Fruit consumption				0.077 ^†^
≥2 portions/day	111 (24.8)	69 (62.2)	42 (37.8)	
<2 portions/day	336 (75.2)	239 (71.1)	97 (28.9)	
Vegetable consumption				0.057 ^†^
≥3 portions/day	60 (13.4)	35 (58.3)	25 (41.7)	
<3 portions/day	387 (86.6)	273 (70.5)	114 (29.5)	
Meat, fish, and eggs consumption				0.478
<3 liangs/day (1 liang = 38 g)	183 (41.0)	122 (66.7)	61 (33.3)	
3–4 liangs/day	213 (47.8)	153 (71.8)	60 (28.2)	
≥5 liangs/day	50 (11.2)	33 (66.0)	17 (34.0)	
Fried food				0.316
<1 time/day	350 (79.4)	246 (70.3)	104 (29.7)	
≥1 time/day	91 (20.6)	59 (64.8)	32 (35.2)	
Sweetened food				0.171
<1 time/day	240 (53.9)	163 (67.9)	77 (32.1)	
1 time/day	161 (36.2)	118 (73.3)	43 (26.7)	
≥2 times/day	44 (9.9)	26 (59.1)	18 (40.9)	
Snack consumption				0.798
<1 time/day	147 (33.2)	101 (68.7)	46 (31.3)	
1 time/day	188 (42.4)	127 (67.6)	61 (32.4)	
≥2 times/day	108 (24.4)	77 (71.3)	31 (28.7)	
Having breakfast everyday				0.186
Yes	350 (79.0)	247 (70.6)	103 (29.4)	
No	93 (21.0)	59 (63.4)	34 (36.6)	
Regular 3 meals/day				0.366
Yes	386 (86.9)	269 (69.7)	117 (30.3)	
No	58 (13.1)	37 (63.8)	21 (36.2)	

^a^ Hypertension was defined as systolic and/or diastolic blood pressure equal to or above the 95th percentile for specific age, gender, and height recommended in the 2018 Chinese Guidelines for Prevention and Treatment of Hypertension for Children [26]. ^b^ Participants with sleep duration less than 9 h for those aged 5–13 years and 8 h for those aged 14–17 years per night were grouped as insufficient sleep [28]. † *p* < 0.10. Abbreviations: MVPA, moderate-to-vigorous-intensity physical activity.

**Table 3:** Associations between background characteristics and hypertension in children with intellectual disability.

	Hypertension ^a^			
	OR_unadj._ (95% CI)	*p*-Value	OR_adj._ (95% CI)	*p*-Value
**Children’s characteristics**				
Gender				
Male	1.00 (Ref.)	0.304	1.00 (Ref.)	0.249
Female	1.25 (0.82, 1.91)		0.78 (0.51, 1.19)	
Birthplace				
Hong Kong	1.00 (Ref.)	0.975	1.00 (Ref.)	0.965
Others	0.99 (0.56, 1.75)		0.99 (0.56, 1.76)	
ID level				
Mild (IQ: 55–69)	1.00 (Ref.)	0.148	1.00 (Ref.)	0.126
Moderate (IQ: 35–54)	1.39 (0.89, 2.16)		1.42 (0.91, 2.21)	
Autism				
No	1.00 (Ref.)	0.401	1.00 (Ref.)	0.582
Yes	0.84 (0.56, 1.26)		0.89 (0.58, 1.36)	
ADHD				
No	1.00 (Ref.)	0.467	1.00 (Ref.)	0.532
Yes	1.17 (0.77, 1.77)		1.14 (0.75, 1.74)	
Down Syndrome				
No	1.00 (Ref.)	0.825	1.00 (Ref.)	0.686
Yes	0.92 (0.46, 1.87)		0.86 (0.42, 1.77)	
Epilepsy				
No	1.00 (Ref.)	0.780	1.00 (Ref.)	0.849
Yes	0.90 (0.42, 1.93)		0.93 (0.43, 2.01)	
Obesity ^b^				
No	1.00 (Ref.)	0.001 **	1.00 (Ref.)	0.001 **
Yes	3.10 (1.62, 5.96)		3.21 (1.66, 6.18)	
**Parental characteristics**				
Paternal education				
Junior secondary and below	1.00 (Ref.)		1.00 (Ref.)	
Senior secondary	1.50 (0.91, 2.48)	0.115	1.46 (0.88, 2.42)	0.091 ^†^
College or above	1.22 (0.72, 2.08)	0.462	1.21 (0.71, 2.06)	0.492
Maternal education				
Junior secondary and below	1.00 (Ref.)		1.00 (Ref.)	
Senior secondary	0.95 (0.59, 1.51)	0.813	0.92 (0.58, 1.48)	0.740
College or above	0.82 (0.48, 1.42)	0.485	0.81 (0.47, 1.40)	0.447
Paternal occupation				
Administrators and professionals	1.00 (Ref.)		1.00 (Ref.)	
Others (clerks, sales representatives, and workers)	1.69 (1.08, 2.63)	0.021 *	1.71 (1.10, 2.68)	0.018 *
Missing	1.90 (0.89, 4.06)	0.095 ^†^	2.00 (0.93, 4.30)	0.074 ^†^
Maternal occupation				
Housewives	1.00 (Ref.)		1.00 (Ref.)	
Administrators and professionals	0.73 (0.42, 1.29)	0.282	0.74 (0.42, 1.30)	0.291
Others (clerks, sales representatives, and workers)	0.78 (0.49, 1.25)	0.303	0.80 (0.50, 1.28)	0.345
Parental marital status				
Married and cohabiting	1.00 (Ref.)	0.082 ^†^	1.00 (Ref.)	0.087 ^†^
Divorced, separated, and widowed	0.60 (0.33, 1.07)		0.60 (0.33, 1.08)	
Paternal obesity ^c^				
No (BMI < 28 kg/m^2^)	1.00 (Ref.)		1.00 (Ref.)	
Yes (BMI ≥ 28 kg/m^2^)	1.15 (0.63, 2.09)	0.657	1.11 (0.61, 2.04)	0.735
Missing	1.03 (0.60, 1.77)	0.913	1.03 (0.60, 1.77)	0.915
Maternal obesity ^c^				
No (BMI < 28 kg/m^2^)	1.00 (Ref.)		1.00 (Ref.)	
Yes (BMI ≥ 28 kg/m^2^)	1.22 (0.55, 2.73)	0.623	1.22 (0.55, 2.74)	0.625
Missing	0.95 (0.48, 1.89)	0.893	0.98 (0.49, 1.94)	0.943

^a^ Hypertension was defined as systolic and/or diastolic blood pressure equal to or above the 95th percentile for age, gender, and height recommended in the 2018 Chinese Guidelines for Prevention and Treatment of Hypertension for Children [26]. ^b^ Child obesity was defined by age- and gender-specific cut-off points of BMI recommended by Cole [27]. ^c^ Parental obesity was defined as BMI ≥ 28 kg/m^2^ for Chinese adults [30]. OR_unadj._, unadjusted odds ratio; OR_adj._, adjusted odds ratio, adjusted for children’s gender and age (continuous variable), except for gender, which was adjusted for age only. † *p* < 0.10; * *p* < 0.05; ** *p* < 0.01. Abbreviations: CI, confidence interval; ID, intellectual disability; IQ, intelligence quotient; ADHD, attention deficit hyperactivity disorder; BMI, body weight index.

**Table 4:** Associations between health-related behaviors and hypertension in children with intellectual disability.

	Hypertension ^a^			
	OR_unadj._ (95% CI)	*p*-Value	OR_adj._ (95% CI)	*p*-Value
Physical activity				
MVPA ≥ 60 min/day	1.00 (Ref.)	0.934	1.00 (Ref.)	0.791
MVPA < 60 min/day	0.97 (0.44, 2.11)		0.89 (0.39, 2.06)	
Sedentary behavior				
<4 h/day	1.00 (Ref.)	0.710	1.00 (Ref.)	0.673
≥4 h/day	0.92 (0.61, 1.41)		1.10 (0.71, 1.72)	
Sleep duration				
Sufficient ^b^	1.00 (Ref.)	0.210	1.00 (Ref.)	0.351
Insufficient ^b^	1.31 (0.86, 1.99)		1.24 (0.79, 1.94)	
Fruit consumption				
≥2 portions/day	1.00 (Ref.)	0.078 ^†^	1.00 (Ref.)	0.262
<2 portions/day	0.67 (0.43, 1.05)		0.76 (0.47, 1.23)	
Vegetable consumption				
≥3 portions/day	1.00 (Ref.)	0.059 ^†^	1.00 (Ref.)	0.159
<3 portions/day	0.59 (0.34, 1.02)		0.65 (0.36, 1.19)	
Meat, fish, and eggs consumption				
<3 liangs/day (1 liang = 38 g)	1.00 (Ref.)		1.00 (Ref.)	
3–4 liangs/day	0.78 (0.51, 1.20)	0.266	0.80 (0.50, 1.28)	0.350
≥5 liangs/day	1.03 (0.53, 2.00)	0.929	1.14 (0.56, 2.35)	0.718
Fried food				
<1 time/day	1.00 (Ref.)	0.317	1.00 (Ref.)	0.251
≥1 time/day	1.28 (0.79, 2.09)		1.36 (0.81, 2.28)	
Sweetened food				
<1 time/day	1.00 (Ref.)		1.00 (Ref.)	
1 time/day	0.77 (0.50, 1.20)	0.250	0.68 (0.42, 1.09)	0.106
≥2 times/day	1.47 (0.76, 2.83)	0.256	1.49 (0.74, 3.00)	0.262
Snack consumption				
<1 time/day	1.00 (Ref.)		1.00 (Ref.)	
1 time/day	1.06 (0.66, 1.68)	0.822	0.95 (0.58, 1.57)	0.853
≥2 times/day	0.88 (0.51, 1.52)	0.656	0.80 (0.45, 1.43)	0.449
Having breakfast everyday				
Yes	1.00 (Ref.)	0.187	1.00 (Ref.)	0.191
No	1.38 (0.86, 2.24)		1.41 (0.84, 2.34)	
Regular 3 meals/day				
Yes	1.00 (Ref.)	0.367	1.00 (Ref.)	0.708
No	1.31 (0.73, 2.33)		1.13 (0.60, 2.11)	

^a^ Hypertension was defined as systolic and/or diastolic blood pressure equal to or above the 95th percentile for age, gender, and height recommended in the 2018 Chinese Guidelines for Prevention and Treatment of Hypertension for Children [26]. ^b^ Participants with sleep duration less than 9 h for those aged 5–13 years and 8 h for those aged 14–17 years per night were grouped as insufficient sleep [28]. OR_unadj._, unadjusted odds ratio; OR_adj._, adjusted odds ratio, adjusted for children’s gender, age (continuous variable), and the background variables with *p* < 0.10 for adjusted ORs in Table 3, including obesity, parental marital status, and paternal occupation. † *p* < 0.10. Abbreviations: CI, confidence interval; MVPA, moderate-to-vigorous-intensity physical activity.

**Table 5:** Summary results of the hierarchical logistic regression of hypertension in children with intellectual disability.

	Hypertension ^a^	
	OR_adj._ (95% CI)	*p*-Value
**Children’s characteristics**		
Gender		
Male	1.00 (Ref.)	0.554
Female	1.16 (0.70, 1.93)	
Age (in years)	0.96 (0.90, 1.02)	0.198
Obesity ^b^		
No	1.00 (Ref.)	0.010 *
Yes	2.77 (1.28, 5.99)	
**Parental characteristics**		
Paternal occupation		
Administrators and professionals	1.00 (Ref.)	0.006 *
Others (clerks, sales representatives, and workers)	2.32 (1.28, 4.19)	
Missing	2.07 (0.46, 9.43)	0.346
Paternal education		
Junior secondary and below	1.00 (Ref.)	
Senior secondary	1.70 (0.94, 3.05)	0.078 ^†^
College or above	2.42 (1.19, 4.94)	0.015 *

^a^ Hypertension was defined as systolic and/or diastolic blood pressure equal to or above the 95th percentile for age, gender, and height recommended in the 2018 Chinese Guidelines for Prevention and Treatment of Hypertension for Children [26]. ^b^ Child obesity was defined by age- and gender-specific cut-off points recommended by Cole [27]. Hierarchical logistic regression was performed with gender and age (continuous variable) forcedly entered in Block 1; the background characteristics with *p* < 0.10 in Table 3 were forward selected in Block 2; health-related behaviors were forward selected in Block 3. *p* = 0.10 and *p* = 0.15 were used as entry and removal criteria, respectively. † *p* < 0.10; * *p* < 0.05. Abbreviation: CI, confidence interval.

## Data Availability

The data that support the findings of this study are available on request from the corresponding author, [Y.G.]. The data are not publicly available due to restrictions (e.g., containing information that could compromise the privacy of research participants).
